# Superlative Feature Selection Based Image Classification Using Deep Learning in Medical Imaging

**DOI:** 10.1155/2022/7028717

**Published:** 2022-09-26

**Authors:** Mamoona Humayun, Muhammad Ibrahim Khalil, Ghadah Alwakid, N. Z. Jhanjhi

**Affiliations:** ^1^Department of Information Systems, College of Computer and Information Sciences, Jouf University, Sakakah, Saudi Arabia; ^2^Department of Computer Science, Bahria University, Islamabad, Pakistan; ^3^Department of Computer Science, College of Computer and Information Sciences, Jouf University, Sakakah, Saudi Arabia; ^4^School of Computer Science and Engineering (SCE), Taylor's University, Subang Jaya, Malaysia

## Abstract

Medical image recognition plays an essential role in the forecasting and early identification of serious diseases in the field of identification. Medical pictures are essential to a patient's health record since they may be used to control, manage, and treat illnesses. On the other hand, image categorization is a difficult problem in diagnostics. This paper provides an enhanced classifier based on the outstanding Feature Selection oriented Clinical Classifier using the Deep Learning (DL) model, which incorporates preprocessing, extraction of features, and classifying. The paper aims to develop an optimum feature extraction model for successful medical imaging categorization. The proposed methodology is based on feature extraction with the pretrained EfficientNetB0 model. The optimum features enhanced the classifier performance and raised the precision, recall, F1 score, accuracy, and detection of medical pictures to improve the effectiveness of the DL classifier. The paper aims to develop an optimum feature extraction model for successful medical imaging categorization. The optimum features enhanced the classifier performance and raised the result parameters for detecting medical pictures to improve the effectiveness of the DL classifier. Experiment findings reveal that our presented approach outperforms and achieves 98% accuracy.

## 1. Introduction

Health informatics has recently emerged as a prominent study area in which computer technology fulfills the needs of human medical demands. Many research efforts have been carried out in image data examination with the goal of diagnostic and clinical investigations [1]. Images are captured via computer-aided investigative analysis using various imaging technologies such as magnetic resonance, computed tomography (CT) scans, and ultrasound [2]. When it concerns image classification issues, both structure in place and discriminative strength of the retrieved features are essential to obtain superior classification results [3]. Medical image collections are utilized for image categorization as well as education [4]. It usually includes photos of distinct attributes depicted under various situations and precise descriptions [5]. It is critical in medical diagnostics to identify the most relevant risk factor with illness identification. Many essential aspects of traditional medical picture categorization methods, such as hue, texture, and size, are present [6]. Classification of images is a critical field that presents a significant difficulty for a visual to be categorized using specialized medical knowledge.

**Figure 1:**
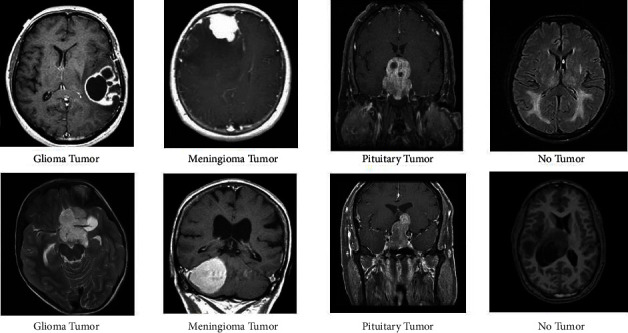
Brain tumor classes.

The brain tumor is among the most dreadful diseases in today's society. One of the most prevalent causes is the systemic change of aberrant cells within the brain, as seen in Figure 1. The tumor size is tiny at the early stage and is referred to as benign in biology. Tumors can spread in the secondary stage from other regions of the body, and their size is bigger than benign, giving rise to the term malignant. Approximately 87,000 people will be detected with brain cancer by 2020 [7]. It is anticipated that in 2021 there will be 84,170 people diagnosed with a brain tumor. There will be 69,950 people diagnosed over the age of 40. Because of the high mortality rate, high-grade glioma and low-grade glioma are the two forms of tumors. Furthermore, low-grade glioma has a higher survival rate than high-grade glioma. Because the survival rate of high grade is roughly 2 years, rapid treatment is essential.

Physicians and doctors can manually evaluate all test findings and photos, but it may take a long time [8]. To improve patient care, improved healthcare innovation in the type of automated instruments is required to promote efficiency [9, 10]. This study aims to provide automated tools to assist clinicians in diagnosing patients to avoid misdiagnosis and prioritize challenging medical diagnoses [11]. This study, in particular, automates the detection of brain tumor kinds using clinical brain images [12, 13]. A radiologist must review several image slices to diagnose health abnormalities in brain imaging, which takes time. Our objective is to correctly identify brain tumors to increase treatment efficiency while leaving the most challenging diagnosis to medical professionals [14].

The study's primary aim is to develop a brain tumor classification method that is accurate, convenient to use, and minimal in complexity. The EfficientNet models were created to minimize available resources while retaining excellent accuracy while spending less memory and training time. Transfer learning methods have been a common supplement to deep learning solutions for categorization challenges. This study investigates the use of pretrained EfficientNets and subsequently fine-tuning for categorizing brain tumors and coupled data enhancement with min-max normalization to improve tumor cell contrast. Furthermore, the inclusion of stain normalization in the preprocessing stage makes the model robust.

This article examines miming the clinical image categorization methods. The DL technique was utilized to decide whether the presented medical imaging was dangerous or healthy. Furthermore, the suggested model predictions were also validated against disease detection and diagnosis methods. Traditional brain tumor classification approaches, prior to object categorization, use a region-based classification of tumors. Based on the results of this study, we provide an automated method for identifying brain tumors using Deep Learning (DL). CNN is composed of a convolutional neural network for feature extraction and classification. The training is carried out using data that is unbalanced. The average pooling layer, which contains detailed characteristics of each tumor type, is taken from the DL model. The remaining paper is organized as follows: Section 2 of the paper presents the literature work.  Section 3 presents the proposed methodology.  Section 4 includes the discussion, and the last section contains the conclusion and future work.

## 2. Literature Review

The brain is the sympathetic nervous organism's command and control center and is in charge of all biological functions. Brain tumors can directly endanger a person's life. When a tumor is found at a preliminary phase, the patient's chances of survival improve [15, 16]. Magnetic resonance imaging (MRI) is commonly used by doctors to identify the presence of cancers or to diagnose tumors. The quality of brain cancer therapy is determined by the doctor's expertise and understanding [17, 18]. As a result, adopting a computerized and flawlessly functioning tumor detection scheme is critical to assisting clinicians in detecting brain cancers [19]. Detecting malignancies in the brain using MR imaging is now an essential job, and various research studies have been undertaken in recent years [20].

Human evaluation is one of the most used methods for brain tumor recognition and characterization from MR images. It relies heavily on the knowledge of physicians who extensively examine the properties of image slices, making it time-taking and vulnerable to inaccuracy [21, 22]. As a result, researchers have expressed a need for the novel technique of computer-aided diagnosis (CAD). As a result, there is a significant chance of building a computerized diagnostic tool that will automatically identify and diagnose brain cancers from MR images, therefore assisting medical professionals [23].

MRI is a nonionizing radiation imaging tool used more often than CT images to identify and treat brain tumors. Determining structures or patterns for identification from highly varied photos takes time and requires much human work, mainly if the data is vast [24, 25]. Several strategies have been presented to assist radiologists in improving their diagnostic accuracy. Neural networks and their generality have just recently emerged [26]. Convolutional Neural Networks (CNNs), which enable learning a hierarchy of more complicated characteristics straight from data, have lately produced excellent achievements in improving medical diagnosing and disease categorization accuracy [27, 28].  Table 1 provides an overview of the existing approaches to better understand the need and motivation of the current study.

So far, the approaches used are generally classified into two types: classic machine learning and DL techniques. For the categorization of a brain tumor, traditional techniques rely on low-grade information and the use of numerical learning methods [36]. This association of segmentation techniques focuses on estimating the tumor's borders and positioning, which includes certain preprocessing procedures like contrast amplification, image smoothing, and edge recognition. In contrast to conventional approaches, deep learning-based techniques rely primarily on training data and need substantially less preparation than traditional alternatives [37]. DL research shows that a system's accuracy highly depends on the data volume.

The approach for creating picture saliency maps is presented in the paper [38]. Saliency maps are created by merging randomized projections with probability density measures to smaller dimensions. This procedure applies to all types of photos. The calculation is efficient owing to the arbitrary projection technique's great processing efficiency; picture resolution is preserved, and the approach is parameterless. We also proved the method's resistance to Gaussian blur picture alteration [38]. The heuristic technique is used to create a hybrid solution for picture classification challenges. The Red Fox Optimization Algorithm was adapted to evaluate images using various fitness measures. The analytical unit, driven by the clustered principle, reviewed the heuristic findings and chose whether or not to crop the picture. CNN categorized such a generated image. The combination of image processing algorithms with key lookup yields excellent results. This method might find the relevant regions, specify where the item is, and then trim it to just the discovered area [39].

CNN-based classification approaches use a three-step methodology to predict the existence or stage of a brain tumor. The initial preprocessing step is eliminating the noise from the MRI and then using algorithms to partition the tumor. The next phase is training, which involves providing the classifier with labels and learned characteristics from every visual in the dataset set for training [40]. The predictor detects the characteristics of several tumor classifications using categorized training data. The testing part employs the same extracting feature approach as the training stage. Based on the learned classifier, these extracted features are provided to the classifier for the definitive classification of the brain tumor class.

For many years, tumors have been examined. On the other hand, most studies look at tumor classification and mean survival estimation separately, oblivious to the intrinsic link between these crucial research tasks. This article provides an innovative automated approach based on DL, which considers the existing challenges of multilabel brain tumor diagnoses, such as resemblance across tumor kinds, a decrease of key characteristics, and feature space repositories. The major goal of this study was to identify brain tumors using MRI scans. To make the procedure more effective, a pretrained DL model was employed to classify the tumors.

## 3. Proposed Methodology

**Figure 2:**
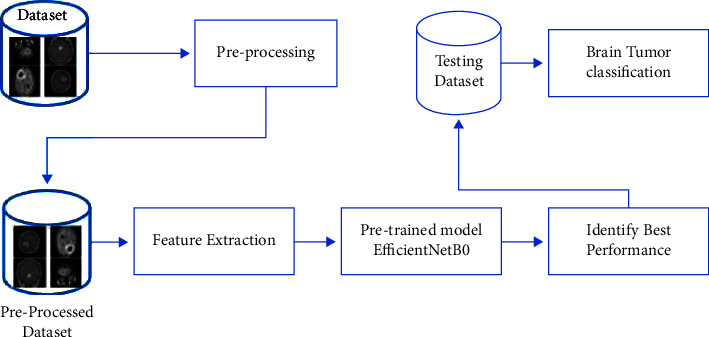
Proposed methodology.

We used transfer learning to apply a pretrained DL network, EfficientNetB0, for brain tumor classification. The architecture of CNNs contains the hierarchy of spatial features, convolutional layers, fully connected layer filters, and pooling layers. Transfer learning takes the information obtained from solving an issue and applies it to solving similar challenges using a trained model to learn new data collection. An image is fed into the networks, which then output the entity label and the probability for each of the categories. In the pretrained network, the last four layers were modified to fit the classification target. The proposed methodology is depicted diagrammatically in Figure 2, where the model output represents the type of tumor.

The development of CNN architectures depends on the available resources, and then the scaling occurs to achieve better performance when there is an increase in resources. Scaling the model has traditionally been done randomly by increasing the CNN depth or the input picture resolution. This method requires time-consuming manual adjustment and sometimes results in improved performance. Trainable weights are part of the CNN architecture. In a convolution layer of the DL model, the preceding layer's in-depth features become acquirable kernels and are used to build the throughput feature map by employing the activation function. Convolutions may be applied to numerous input maps to create a single outcome map. Spatial and high-level properties are learned using weights. Each layer takes a measure as an input and outputs extracted features. Gradient descent and backpropagation are used to execute the learning process, which is stated as follows:

(1) $$\begin{array}{c} \delta_{p}^{q}=R\left(\beta_{p}^{q}+\overset{N^{q-1}}{\underset{r=1}{\sum }}w_{p,r}^{q}\times M_{r}^{q-1}\right)\text{,} \end{array}$$

where *δ*_*p*_^*q*^ shows the layer output, bias represented as *β*_*p*_^*q*^, layer weight represented as *w*_*p*,*r*_^*q*^, layer inputs represented as *M*_*r*_^*q*−1^, and ReLU (R) is used as the activation function. The weights are adjusted by utilizing the preceding layer's results as a feed to the following layer.

(2) $$\begin{array}{c} R\left(M\right)=\max\;\left(0,M\right)\text{.} \end{array}$$

ReLU function transforms negative values to zero, and the max-pooling layer computes the matrix's maximum weights.

(3) $$\begin{array}{c} \delta_{p}^{q}\left(\;\max\;\right)={Pool}_{\max}\left(Rel\left(M\right)\right),Rel\left(M\right)\in \delta_{p}^{q}\text{.} \end{array}$$

The stride value is used to shift each matrix. This layer's properties are the filter dimensions and stride. The softmax *f*(*s*)_*i*_ is used as the activation function.

(4) $$\begin{array}{c} f{\left(s\right)}_{i}=\frac{e^{\delta_{p}^{q}}}{\overset{n}{\underset{n=1}{\sum }}e^{\delta_{p}^{q}}}\text{.} \end{array}$$

For multiple classes, the categorical cross-entropy (CE) loss function is used that calculates the loss.

(5) $$\begin{array}{c} CE=\overset{n}{\underset{i}{\sum }}z_{i}\;.\;\log{\hat{z}}_{i}\text{,} \end{array}$$

where ${\hat{z}}_{i}$ is the output. Its purpose is to calculate the distinction between different probability distributions. Further to the extraction and selection of features, the classifying process utilizing DNN is executed on the resulting feature space.

**Figure 3:**
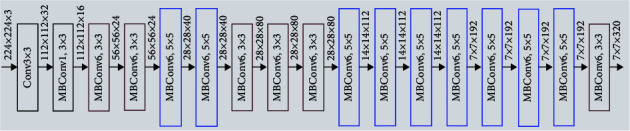
EfficientNetB0.

The EfficientNet models from EfficientNetB0 to EfficientNetB7. Scaling the model increases model performance by optimizing the architectural width, depth, and picture resolution compound parameters. The EfficientNetB0 structure is shown in Figure 3. The EfficientNet model series was constructed using a neural architecture to generate a baseline model. The layer mobile inverted bottleneck (MBConv) is the block of the EfficientNet model. EfficientNet outperforms all antecedents with comparable compute and memory requirements by a wide margin.

**Figure 4:**
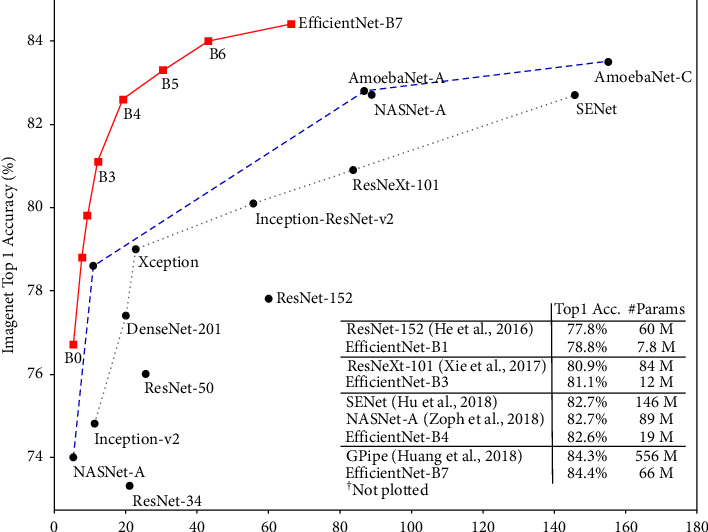
Number of parameters (millions) [41].

The EfficientNet frameworks are based on simple and incredibly efficient compounding scaling algorithms. This method enables you to grow exponentially the ConvNet foundation to any target-constrained capacity while keeping the network utility utilized for training dataset transition. In general, EfficientNet variants outperform existing CNNs such as AlexNet, GoogleNet, and MobileNetV2 as shown in Figure 4. EfficientNet has many variants, each with a different set of parameters ranging from 5.3 million to 66 million. Learning may be effective for categorization issues, but all of the additional new challenges need whole unique classes. EfficientNetB0 contains 5.3 million parameters and 0.39 billion flops.

## 4. Experiment and Results

**Figure 5:**
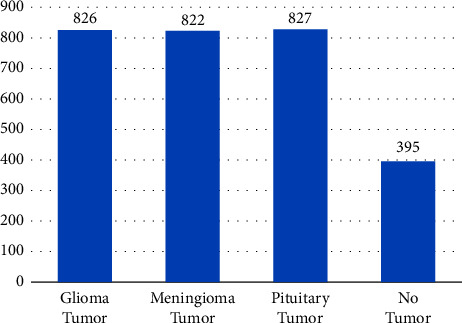
Training data distribution.

**Figure 6:**
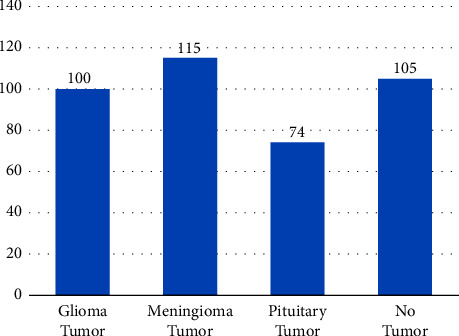
Testing data distribution.

The network was trained and tested using an MRI images database obtained from an online Kaggle platform [42]. The training dataset contains glioma tumor 826, meningioma tumor 822, pituitary tumor 827, and 395 images with no tumor, as shown in Figure 5. The testing dataset contains glioma tumor 100, meningioma tumor 115, pituitary tumor 74, and 105 images with no tumor, as shown in Figure 6. In the preprocessing phase, we normalize the MRI images.

The proposed network has been trained on 50 epochs. The pretrained model weights of EfficientNetB0 on the ImageNet dataset were utilized by initialization and fine-tuned further. The dataset is segmented with several transformations to avoid model overfitting. While training, the ADAM optimizer and loss function categorical cross-entropy are used. Excessive epochs can contribute to the overfitting of the training data; however, insufficient epochs might lead to an underfit framework. Earlier stopping is a technique that enables you to provide an arbitrarily large range of training epochs and then stop training when the network's output on stand-out validation data ceases to increase. The following evaluation metrics evaluate the results.

(6) $$\begin{array}{c} Accuracy=\frac{TP+TN}{TP+FP+FN+TN}\text{,}\\ Precision=\frac{TP}{TP+FP}\text{,}\\ Recall=\frac{TP}{TP+FN}\text{,}\\ F1Score=2*\frac{Precision*\;Recall}{Precision+Recall}\text{.} \end{array}$$

Data augmentation was conducted before training to improve the accuracy rate. The number of True Positive, True Negative, False Positive, and False Negative pixels is represented by TP, TN, FP, and FN, respectively.

**Figure 7:**
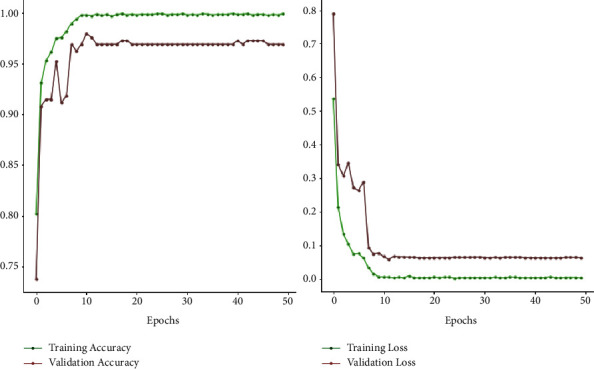
Graphical presentation of accuracy and loss using model EfficientNetB1.

**Figure 8:**
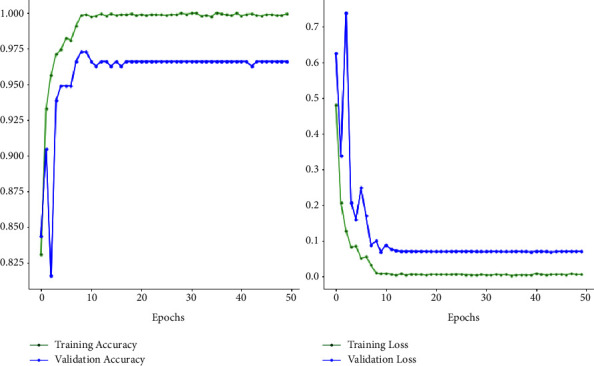
Graphical presentation accuracy and loss using model EfficientNetB0.

**Figure 9:**
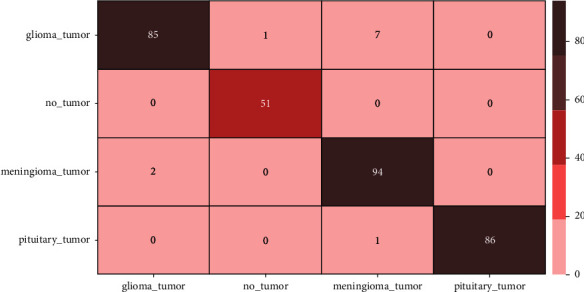
Confusion matrix using model EfficientNetB1.

**Figure 10:**
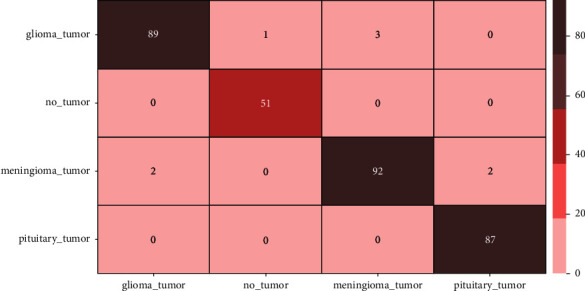
Confusion matrix using model EfficientNetB0.

Figures 7 and 8 depict the accuracy and loss variability overtraining and validation procedure throughout the model learning process with EfficientNetB0 and EfficientNetB1 DL models. Figures 9 and 10 represent the confusion matrix that determines the classification performance of every class. The model results are shown in Table 2. The proposed approach achieves 98% accuracy.

**Figure 11:**
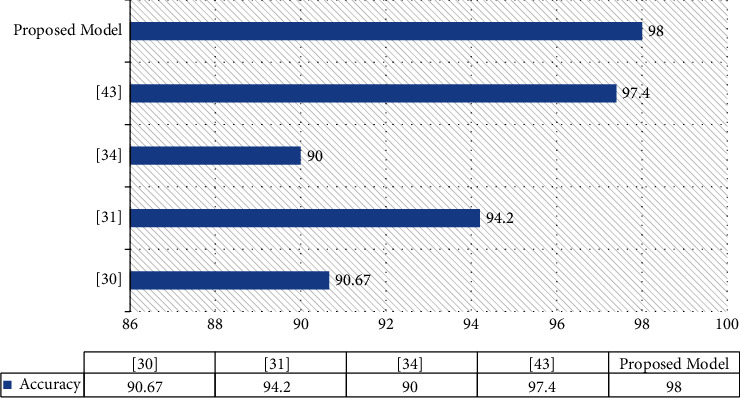
Results comparison.

We conducted experiments on a dataset of brain tumor MRI images that were separated into training and testing segments. The data augmentation was used to improve our dataset by making minor modifications to our MRI pictures and extracting these enhanced images from our proposed CNN model. With a batch size of 32, we trained the model for 50 epochs. The experiment was carried out in Python using TensorFlow and Keras packages. We studied the number of parameters by fine-tuning a pretrained model. In contrast to the existing methods, our proposed model yields good MRI results that employ various data augmentation strategies as shown in Figure 11. We conducted an experiment that used the brain tumor dataset to attain the greatest accuracy of 98% employing the fine-tuned EfficientNetB0 model. The comparatively strong performance of the CNN, also with limited datasets, demonstrates the potential of CNN feature representation.

## 5. Discussions

We presented an automated brain tumor classification approach. The model performance is dependent on accurate feature extraction and appropriate metric learning. We retrieved the features using transfer learning capabilities and fine-tuning the CNN model. We conducted this study using the EfficientNetB0 model since its framework is more extensive and ideal for feature extraction in aspects of localization and identification of particular information from the data. Small datasets, especially in healthcare imaging, necessitate the use of pretrained DL models with the transfer learning approach. The bias is in the model's performance indicators, which are skewed due to the possibility of overfitting. The facts we see are affected not just by the substantial relationships you are seeking to model, but also by a variety of additional elements unique to the circumstances surrounding the collection of this dataset. The modeling may use these characteristics, but the resultant predictive ability is attributed to predictor factors. Validating and testing using entirely different datasets allow for some of such uncertainty to be accounted for in the proposed model.

The study's limitation is its restricted emphasis on brain tumors, which may be expanded to account for diverse forms of cancer threats in MRI imaging. This may involve using a range of imaging modalities and classification approaches to get the finest forecast of damaged areas in the brain and separate these regions from undamaged portions of the brain. Various image identification methodologies might be used to show the missing picture characteristics in the stabilized image and perform the finest classification results.

CNNs have performed well in computer vision on labeled datasets like ImageNet which includes over one million labeled photos. The adoption of a frequency spectrum data augmentation approach in preprocessing has resulted in improved picture quality and efficiency of brain tumor assessments [44]. The training time increases steadily as the number of iterative epochs increases, implying that we may save time by employing fewer epochs. On the other hand, the number of iterations or the depth architectures has no effect on classification accuracy. CNN's great accuracy allows the physician to pinpoint the source, size, and severity of tumors, which aids in constructing a successful treatment strategy.

## 6. Conclusion and Future Work

The classification of brain tumors is critical in clinical applications to establish an appropriate therapy. Medication errors in brain tumors will result in incorrect medical intervention and diminish patients' chances of survival. The proper identification of a brain tumor is critical for optimal treatment strategy in curing and enhancing the lives of individuals with brain tumors. CAD diagnosis systems with CNNs have been successful and have made significant advances in the area of deep learning. The proposed technique is based on feature extraction using the pretrained EfficientNetB0 model. The optimal features improved classifier performance by enhancing precision, recall, F1 score, accuracy, and identification of brain tumors. Experiment results show that our proposed method outperforms and achieves a 98% accuracy. In the future, the model's implementation may include ensemble classifiers to boost the accuracy rate, as well as an expansion to a classification for mode fine-tuning and freezing layers.

## Figures and Tables

**Table 1:** Existing approaches.

Article	Year	Model	Classification classes	Data size
[29]	2020	VGG-16 and VGG-19	T1, T1CE, T2, and Flair	75 low-grade gliomas and 210 high-grade gliomas
[30]	2019	VGG-19	Glioma grades	3064 images of 233 patients
[31]	2019	2D CNN with genetic algorithm	(Meningioma, glioma, and pituitary) and (glioma grades)	600 MRI images
[32]	2019	Customized CNN	Tumor or normal	330 MRI images
[33]	2019	ResNet34	Tumor or normal	48 3D images
[34]	2019	Customized CNN	Glioblastoma, metastatic bronchogenic, and sarcoma	66 MRI images
[35]	2018	VGG-16	Classification of brain tumor type	43 3D images

**Table 2:** Proposed model results.

Model	Glioma tumor				Meningioma tumor				Pituitary tumor				No tumor				Accuracy
	Precision	Recall	F1 score	Support	Precision	Recall	F1 score	Support	Precision	Recall	F1 score	Support	Precision	Recall	F1 score	Support	
EfficientNetB1	0.98	0.91	0.94	93	0.92	0.98	0.95	96	1.0	0.99	0.99	87	0.98	1.0	0.99	51	97
EfficientNetB0	0.98	1.0	0.99	93	0.97	0.96	0.96	96	0.98	1.0	0.99	87	0.98	1.0	0.99	51	98

## Data Availability

The data used to support the findings of this study are available from the corresponding author upon request.
